# History, epidemiology and diagnostics of dengue in the American and Brazilian contexts: a review

**DOI:** 10.1186/s13071-018-2830-8

**Published:** 2018-04-24

**Authors:** Tiago Souza Salles, Thayane da Encarnação Sá-Guimarães, Evelyn Seam Lima de Alvarenga, Victor Guimarães-Ribeiro, Marcelo Damião Ferreira de Meneses, Patricia Faria de Castro-Salles, Carlucio Rocha dos Santos, Ana Claudia do Amaral Melo, Marcia Regina Soares, Davis Fernandes Ferreira, Monica Ferreira Moreira

**Affiliations:** 1 Departamento de Bioquímica, Instituto de Química, Universidade Federal do Rio de Janeiro, Rio de Janeiro, RJ, 21941-909 Brazil; 2 Departamento de Virologia, Instituto de Microbiologia, Universidade Federal do Rio de Janeiro, Rio de Janeiro, RJ 21941-590 Brazil; 3 Amil, Americas Medical City, Hospital Vitória, Pediatria, Rio de Janeiro, RJ 22775-001 Brazil; 4 Universidade Federal do Rio de Janeiro, Instituto de Bioquímica Médica, Rio de Janeiro, RJ 21941-902 Brazil; 5 Instituto Nacional de Ciência e Tecnologia em Entomologia Molecular, Rio de Janeiro, 21941-902 RJ Brazil; 6 Department of Molecular and Structural Biochemistry, North Carolina State University, 120 W Broughton Dr, Raleigh, NC USA

**Keywords:** Dengue virus, Dengue history, Dengue epidemiology, Diagnostics of dengue, Serotyping

## Abstract

Dengue virus (DENV), an arbovirus transmitted by mosquitoes, has become a major threat to American human life, reaching approximately 23 million cases from 1980 to 2017. Brazil is among the countries most affected by this terrible viral disease, with 13.6 million cases. DENV has four different serotypes, DENV1-4, which show a broad clinical spectrum. Dengue creates a staggering epidemiological and economic burden for endemic countries. Without a specific therapy and with a commercial vaccine that presents some problems relative to its full effectiveness, initiatives to improve vector control strategies, early disease diagnostics and the development of vaccines and antiviral drugs are priorities. In this study, we present the probable origins of dengue in America and the trajectories of its spread. Overall, dengue diagnostics are costly, making the monitoring of dengue epidemiology more difficult and affecting physicians’ therapeutic decisions regarding dengue patients, especially in developing countries. This review also highlights some recent and important findings regarding dengue in Brazil and the Americas. We also summarize the existing DENV polymerase chain reaction (PCR) diagnostic tests to provide an improved reference since these tests are useful and accurate at discriminating DENV from other flaviviruses that co-circulate in the Americas. Additionally, these DENV PCR assays ensure virus serotyping, enabling epidemiologic monitoring.

**Electronic supplementary material:**

The online version of this article (10.1186/s13071-018-2830-8) contains supplementary material, which is available to authorized users.

## Background

Dengue is a disease caused by an arbovirus. This term refers to arthropod-borne viruses, as defined by the World Health Organization (WHO). Dengue virus (DENV) belongs to the family *Flaviviridae* and the genus *Flavivirus* and has four different serotypes (DENV1-4) [1, 2]. This pathogen is an enveloped virus with icosahedral symmetry and a diameter of approximately 50 nm [3, 4]. DENV has a genome consisting of a single positive-polarity RNA strand approximately 10.8 kb in length with an open reading frame that encodes a single polyprotein that is cleaved into the capsid (C), membrane (M), and envelope (E) structural proteins and eight non-structural (NS) proteins, NS1, NS2A, NS2B, NS3, NS4A, NS2K, NS4B and NS5. Structural glycoprotein E is responsible for cell recognition and for promoting entry, which is mediated by a fusion process between the viral envelope and the cell membrane, while the NS proteins aid viral genome replication [2, 3, 5].

Dengue fever (DF) has been well studied because it is a systemic and dynamic infection with a broad clinical spectrum that includes serious and non-serious clinical manifestations. This infection can evolve in phases: a feverish, critical phase with hemorrhagic fever symptoms, known as dengue hemorrhagic fever (DHF), and dengue shock syndrome (DSS). Both phases are considered complications, and uncomplicated cases are characterized by mild, spontaneous or induced manifestations, defined by thrombocytopenia (platelet count < 100,000/mm^3^) followed by a recovery phase [6].

No effective therapy for dengue exists. Treatment is purely symptomatic, requiring a high level of patient care; patients can be hospitalized to facilitate fluid replacement and blood transfusion when indicated [7]. Severe cases occur in approximately 500,000 people/year and present a mortality rate of up to 10% for hospitalized patients and 30% for non-hospitalized patients [5, 8].

DENV and other arboviruses that co-circulate in the Americas, such as Zika virus (ZIKV), yellow fever virus (YFV) and chikungunya virus (CHIKV), are transmitted by mosquito species of genus *Aedes* [9–11], particularly *Aedes aegypti* in the Americas [12].

Dengue in the Americas has an endemo-epidemic pattern with outbreaks every three to five years. Based on the epidemiological patterns of the disease, mainly determined by the reported circulation of the DENVs and the main vector, Brathwaite Dick et al. [13] documented four important time periods of dengue incidence in the Americas from 1600 to 2010. In the Americas, 1 million cases occurred in the 1980s, and 4.7 million cases occurred from 2000 to 2007 [13].

The main objective of this review is to show that the trend toward elevated dengue incidence and number of deaths was even higher in the period 2011–2017, than that in the period 2001–2010. This review aims to provide the reader with knowledge of the disease trajectory, prevention mechanisms and disease etiology, as dengue has become a major public health problem in the Americas, especially in Brazil from 2011 to 2017. Increasingly intense efforts are being made to seek effective vaccines and antiviral agents that can prevent and control this infection. Major problems are the sensitivity and cost of diagnostic tests, which hamper the immediate treatment of symptoms, as well as the high costs of healthcare for dengue patients and the epidemiological monitoring of the disease.

## A brief history of dengue in the Americas

Historically, it has not been determined when DENV first appeared in human populations, mainly because the disease is often asymptomatic and is therefore not diagnosed [2]. The earliest record of dengue comes from a Chinese medical encyclopedia dating back to 992 BC [14]. Moreover, before the end of the 18th century, intermittent epidemics of a specific disease with a strong similarity to dengue occurred in Asia and the Americas; therefore, there is a hypothesis that between the 19th and 20th centuries, the virus probably spread throughout the tropics and subtropics [1].

The trajectory of dengue outbreaks in the Americas was characterized by Brathwaite Dick et al. [13], who described an outbreak history from 1600 to 2010 that was categorized into four phases: introduction of dengue in the Americas (1600–1946), continental plan for the eradication of *Ae. aegypti* (1947–1970) marked by the successful eradication of the mosquito in 18 continental countries, *Ae. aegypti* reinfestation (1971–1999) caused by the failure of the mosquito eradication program, and increased dispersion of *Ae. aegypti* and DENV circulation (2000–2010) characterized by a marked increase in the number of outbreaks [13, 15].

In the Americas, a new outbreak during the period 2011–2017 was coincidentally observed after the great movement of people caused by four global sporting events: the 2011 Pan-American Games in Guadalajara (Mexico), the 2013 Confederations Cup, the 2014 World Cup and the 2016 Olympics, all of them in Brazil. This outbreak was reflected in the dengue mortality rate [16].

Considering data from the Pan-American Health Organization (PAHO) [17], a total of 1,073,978 cases of dengue, 19,450 severe dengue cases and 758 deaths were reported in the Americas at the end of 2011, with an average dengue incidence of 113 cases/100,000 inhabitants [18]. Despite the historical increase in the dengue case number reported in 2011, the number of cases, severe dengue cases, and deaths were reduced by approximately 45% compared to those in 2010. However, the case fatality rate (CFR) maintained the same value (0.07%). The Southern Cone subregion reported a total of 796,548 cases, contributing 71% of the total cases. Surprisingly, Brazil contributed 95% of the total number of cases in this region (756,720 reported cases).

In the final epidemiological week (EW) of 2012, a total of 1,162,998 cases had been recorded in the entire continent, at an average incidence of 120.7 cases/100,000 inhabitants [18]. The data exceeded the total number of cases reported in 2011, with a great increase (approximately 60%) in the total number of severe cases (32,408) and a slight increase (approximately 7%) in the total number of deaths (807) compared to the year 2011. The Southern Cone subregion reported a total of 639,348 cases, contributing 54% of the total cases. Brazil again contributed 93% of the total number of cases in this region (594,593 reported cases).

By the end of 2013, there was an increase in the number of cases; 2,384,234 cases of dengue and 1,403 deaths were reported. The average incidence of dengue was 245 cases/100,000 inhabitants [18]. However, it is important to emphasize that although there was an increase in the number of dengue cases, the total number of severe cases (37,692) and the CFR remained close to the values observed in 2012. The Southern Cone subregion reported a total of 1,627,453 cases, contributing 68% of the total cases. Again, Brazil contributed of the highest number of cases in this region, 1,473,645 cases, contributing 90% of the total cases.

In 2014, there was a decrease in the total cases of dengue to 1,171,029; 15,744 severe dengue cases and 803 deaths were reported. The average incidence of dengue was 194 cases/100,000 inhabitants [18]. Despite the historical increase in the number of reported cases of this disease, an approximately 50% reduction in the number of cases, severe dengue cases, and deaths was reported in 2014 compared to the year 2013. However, the CFR maintained the same value (0.06%).

In 2015, there was an increase in the number of cases; a total of 2,430,178 dengue cases with 1354 deaths were recorded in the entire continent, for an average incidence of 245 cases/100,000 inhabitants [18]. The recorded data exceeded the total number of cases reported in 2014. However, it is important to emphasize that despite the increase in the number of cases, the total number of severe cases (12,824) and the CFR (0.05) remained well below the values observed during 2014. Additionally, Brazil, Colombia and Mexico reported the simultaneous co-circulation of all four serotypes of DENV. The Southern Cone subregion reported a total of 1,054,188 cases, contributing 87% of the total cases on the continent, followed by the Andean subregion and the North and Central America subregion, contributing 6% of the total cases. Brazil contributed 85% of the total number of cases on the continent (896,059 reported cases).

In 2016, there was a decrease in the number of cases; a total of 2,168,146 cases of dengue, 4,366 severe dengue cases and 903 deaths were reported, for a CFR of 0.04%. The average incidence of dengue was 219 cases/100,000 inhabitants [18]. The Southern Cone subregion reported a total of 1,651,575 cases, and Brazil contributed 91% of the total number of cases (1,502,933 reported cases).

In 2017, despite the historical decrease in the reported number of cases of this disease, a total of 577,697 cases, 4,366 severe dengue cases and 903 deaths were reported, and the CFR was maintained at 0.05%. The average incidence of dengue was 58.02 cases/100,000 inhabitants [18]. Brazil, Colombia and Guatemala reported the simultaneous co-circulation of all four serotypes of DENV. The Southern Cone subregion reported a total of 254,453 cases, contributing 43% of the total cases, and Brazil contributed 99% of the total number of reported cases (252,054 cases). Brazil also reported the co-circulation of other arboviruses, such as CHIKV, ZIKV and YFV.

It is important to emphasize that in the 2011–2017 period, there was a significant increase (approximately 30%) in the number of dengue cases, totaling 10,851,043 compared with the 2001–2010 period, which had a total number of 7,641,334 dengue cases [17]. When compared to the previous decades (1980–2017), this last seven-year period contributed 47% of the total number of cases. In according PAHO data the dengue incidence rates over the decades (1980–2017) [18], and the dengue mortality rates of countries and territories in the Americas in the same period, showing that mortality rates were significant over the last ten years (2007–2017) [16].

## History of dengue in Brazil

The earliest reference to dengue in Brazil was made during the colonial period. The first case was described in the city of Recife, Brazil, in 1685. Seven years later, in Salvador, a dengue epidemic led to 2000 deaths. The 1846 dengue outbreak was also considered an epidemic, reaching several states, such as Rio de Janeiro and São Paulo. Between 1846 and 1916, São Paulo was hit by several dengue epidemics [19].

At the beginning of the 20th century, Oswaldo Cruz implemented a mosquito control program that lasted for many years. The great challenge of the time was the yellow fever epidemic [7, 19].

**Fig. 1:**
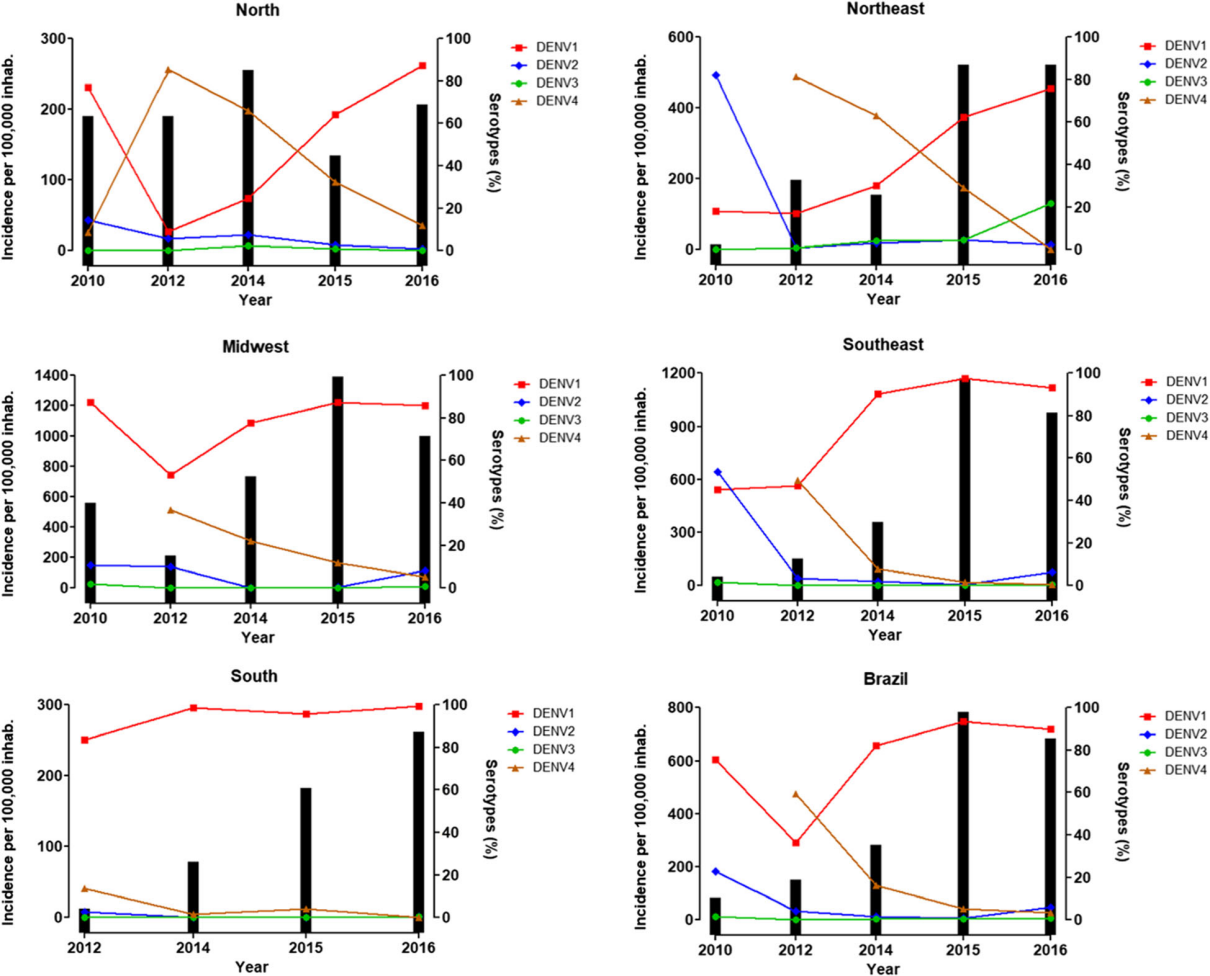
Dengue incidence associated with DENV serotypes in Brazil from 2010 to 2016. Bars: dengue incidence per 100,000 inhabitants related to the left Y-axis. Lines: DENV serotype percentage observed in samples analyzed by LACENs related to the right Y-axis. Calculations were performed for each region for every year using data available from the Brazilian Ministry of Health. 2010 data: up to the 9th week of monitoring. 2016 data: up to the 27th week of monitoring

*Aedes aegypti* was eradicated in Brazil in the 1950s, but it returned in the 1980s. Since the first epidemic of dengue in Roraima, *Ae. aegypti* has persisted in Brazil to the present [7]. During the Roraima epidemic, serotypes DENV1 and 4 were isolated. In 1986, a dengue epidemic arose in Rio de Janeiro and in some urban areas of Northeast Brazil, with DENV1 dissemination and reports of more than 50,000 cases. In 1990, DENV2 was introduced in Rio de Janeiro, reaching several areas of Southeast Brazil. In 1998, a pandemic of more than 500,000 cases occurred in Brazil [7]. The virus spread throughout the country, with the highest number of cases being reported in Northeast Brazil. In 2000, DENV3 was isolated in Rio de Janeiro, and a new dengue epidemic occurred between 2001 and 2003. Several southern states were affected by dengue for the first time, with most cases occurring in people over 15 years of age. In fact, the disease generally affects young adults due to increased exposure, although it may also occur in children [7, 19]. The number of dengue cases and incidence/100,000 inhabitants in different Federative Units (FU) from regions of Brazil from 2011 to 2017 are summarized in Additional file 1: Table S1 (see also Fig. 1). The pattern of dengue endemo-epidemic outbreaks occurring every three to five years was maintained in Brazil until 2010 [13], when it changed to every two years (Additional file 1: Table S1), perhaps because of the movement of people for global sport events in the country.

## Serotypes circulating in Brazil in the 21st century

The monitoring of dengue cases is performed using suspected blood samples through viral isolation followed by the gold standard polymerase chain reaction (PCR) assay [18] used in Brazilian State Central Reference Laboratories (LACENs) for serotyping, allowing evaluation of circulating DENV serotypes. Monitoring reports demonstrated that DENV3 predominated in several Brazilian FU between 2002 and 2006. From 2007 to 2009, a shift was observed, and DENV2 replaced DENV3 as the predominant serotype. This change led to epidemics in several FU, with a rise in severe cases among individuals under 15 years old [7]. The monitoring of circulating serotypes throughout 2009 revealed a new change in the predominant serotype, with a significant recirculation in DENV1, which became the predominant serotype in the FU of Roraima, Mato Grosso do Sul and Piauí beginning in 2008 [7].

In 2010, DENV monitoring in Brazil indicated the circulation of the DENV1, DENV2 and DENV3 serotypes (Fig. 1). However, notably, compared with the other serotypes, DENV1 had the highest incidence in Brazil, except for the Northeast region (which had fewer than 200 cases/100,000 inhabitants) (Fig. 1). This serotype probably directly affected the epidemiological FU situation with epidemic occurrence [7].

The recirculation of DENV1 was a potentially dangerous situation as it could lead to a large disease outbreak in the Brazilian FU since most of the population had never been in contact with this serotype before; DENV1 appeared before the first decade of the 21st century. With DENV2 circulation, an increase in the proportion of severe cases of the disease was also observed, particularly in children and adolescents, including a higher demand for hospital admissions [7]. Indeed, data available from Brazilian Government surveillance agencies revealed that an increase in DENV1 serotype was reported in 2010 and the following years, with a similar increasing pattern of dengue incidence (Fig. 1). The repercussions of DENV1 recirculation should be monitored closely by dengue surveillance at all levels of the health system because this event could cause serious epidemic disease due to the low circulation of this serotype in recent decades [7].

**Fig. 2:**
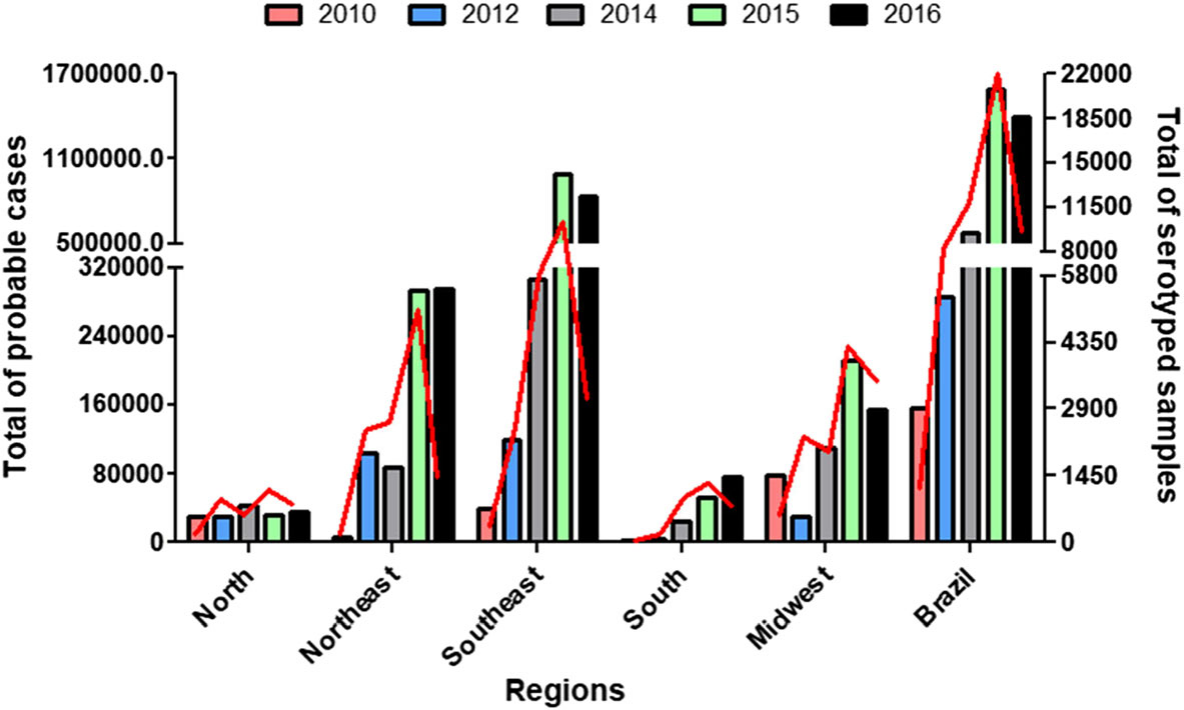
Total probable dengue cases per region of Brazil associated with serotyped samples. Bars: total probable cases related to the left Y-axis. Lines: total serotyped samples related to the right Y-axis. Calculations were performed for each region for every year using data available from the Brazilian Ministry of Health. 2010 data: up to the 9th week of monitoring. 2016 data: up to the 27th week of monitoring

On July 30, 2010, the first suspected case of the DENV4 serotype was collected by the Roraima LACEN, and the sample was sent to the Evandro Chagas Institute (ECI) in accordance with the protocol established by the Ministry of Health. This finding was significant because since DENV4 was identified 28 years ago in Brazil, this serotype had not been observed or detected for a long period. The ECI confirmed a total of three DENV4 dengue cases in Roraima, located in the North Brazilian region (Fig. 2) [20]. Furthermore, during the DENV1 outbreak in March 2011, seven patients with the DENV4 serotype were detected in Niterói City in Rio de Janeiro, indicating the co-circulation of DENV1 and 4 in Brazil [21]. The presence of DENV4 provided the risk not only for the development of more severe manifestations (such as DHF) in people previously infected with DENV1, 2 or 3 but also the possibility of increasing cases in subsequent years. Indeed, except for the Southern region, all the other Brazilian regions presented a rapid increase in DENV4 serotype incidence with a new reduction from 2014 (Fig. 1).

In 2015, a new recurrence of DENV2 was observed, especially in the Northeast region, leading to 1365 cases of severe dengue fever and 18,619 cases of DF in Brazil. Despite the increase in DENV2 serotype infections compared to the same period in 2015, DENV2 represented 6.4% of the total of 2,204,000 cases of the disease registered between January and June 2016. During the same period, 318 deaths related to dengue were confirmed. The data from the last Epidemiological Bulletin of the Ministry of Health showed that by the 49th EW of 2016, 826 cases of severe dengue fever and 8166 dengue fever cases had been confirmed [22]. In the same period in 2015, there were 1680 cases of severe dengue fever and 21,155 cases of dengue fever [22, 23].

The 2016 bulletin confirmed 609 deaths by dengue, representing 6.8% of the total cases. Although the number of dengue deaths was lower in 2016 than in 2015, 972 deaths were confirmed. The data also highlight that 2016 had the second-highest number of dengue cases in Brazil since 1990, when data began to be recorded in Brazil, followed only by 2015 [23]. Notably, as a percentage of the total number of cases, 2015 deaths were lower (4.3% of total cases) than those of 2016. This finding is possibly related to the high number of people infected with DENV1 who may have also become infected with DENV2 in recent years (Fig. 2), increasing the number of serious cases. Once infected by one serotype, a patient is immune only to that specific serotype; however, there is an increased risk of presenting a more severe manifestation of the disease during a second infection with another serotype [23]. Currently, all four serotypes circulate throughout the year in Brazil. When the detection of one serotype increases in a certain region, the effect is usually regional and could remain limited to that region, depending on both the geographic location and the population movement in the area (for example, if many individuals commute to the city for work). Furthermore, epidemics sometimes target regions that were unaffected by the last epidemics of a certain serotype, so that the population is susceptible but the municipalities around it are not susceptible.

**Fig. 3:**
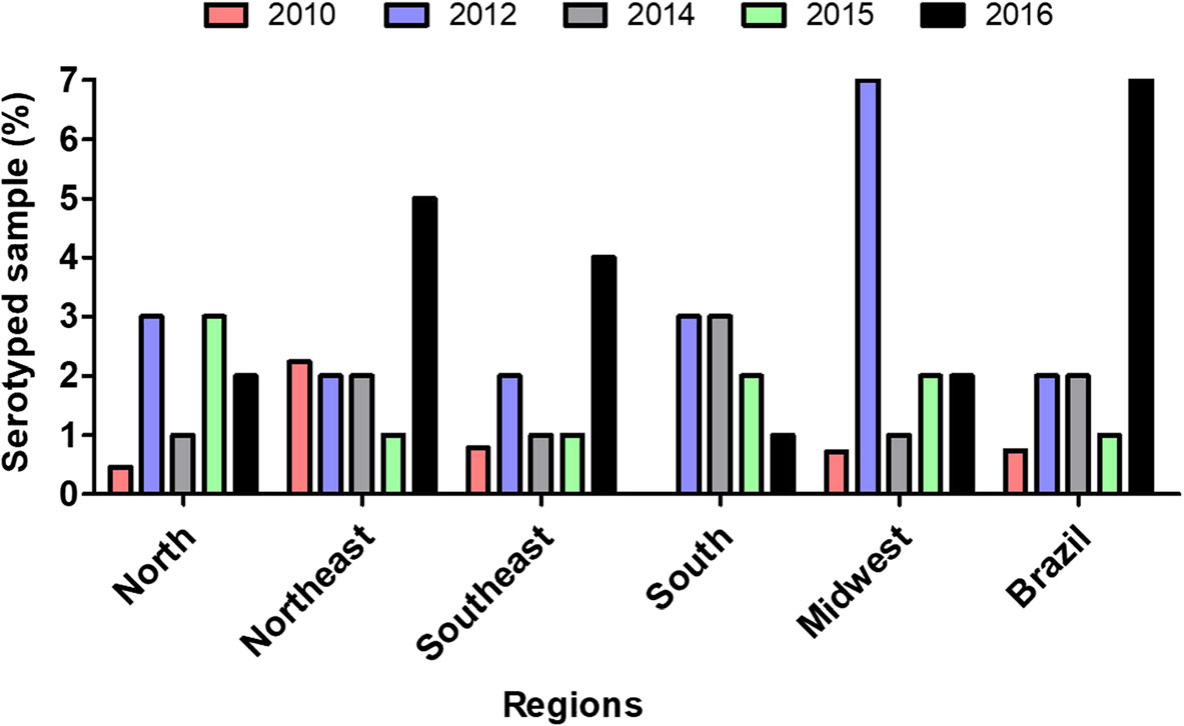
Serotyped samples (%) in several regions of Brazil from 2010 to 2016. The total percentage of serotyped samples was calculated in relation to the total probable cases observed in each region for every year using data available from the Brazilian Ministry of Health. 2010 data: up to the 9th week of monitoring. 2016 data: up to the 27th week of monitoring

Laboratory monitoring is vital for the assessment of circulating serotypes, which provides Brazilian Government agencies with important data to establish strategies focused on combating dengue through vector control aiming to minimize potential disease outbreaks. Figure 2 shows that the increase in dengue serotyping is proportional to the number of cases of dengue reported in different regions, although the serotyping percentage is still lower compared with the total cases. A maximum 7% of total dengue cases were serotyped from 2010 to 2016; this insufficient serotyping percentage clearly underestimates the actual number of cases of dengue that occurs in Brazil (Fig. 3) [7, 19, 22, 24–29] making it difficult to monitor DENV throughout the country. After 2010, some strategies such as laboratory accreditation (LACENs) and greater funding were adopted to further expand the dengue serotyping coverage. Despite the investment, these efforts were not sufficient to increase the percentage beyond 7%, considering the elevated number of dengue cases that occurred in Brazil in 2015 and 2016 (Fig. 3).

## Diagnosis of dengue virus

Dengue, a neglected tropical disease, was recognized as a major public health problem in June of 1999 by being formally included in the disease portfolio of the United Nations Development Programme/World Bank/World Health Organization Special Programme for Research and Training in Tropical Diseases by the Joint Coordination Council [30, 31]. The WHO recommends the use of serological methods for the diagnosis of DENVs to provide laboratory confirmation and the use of genomic techniques for the confirmatory test necessary to directly detect the virus. Currently, three basic steps in DENV detection are followed by most diagnostic laboratories: (i) isolation by animal cell culture; (ii) viral characterization by the detection of specific antigens (NS1 antigen capture enzyme-linked immunosorbent assay, ELISA) and the indirect detection of immunoglobulin M (IgM), IgG, and virus-specific antibodies in serum; and (iii) detection of the DENV genomic sequence using a nucleic acid amplification assay based on PCR, with reverse transcription (RT-PCR) and quantitative PCR (qPCR) being the appropriate tools for the identification and determination of different serotypes of dengue [6].

The Brazilian Ministry of Health recommends the use of the following protocol. All patients suspected of DF must be reported and have a blood sample collected for diagnostics. Up to eight days (preferably five) after the onset of symptoms, patient samples are processed for dengue using ELISA for NS1 detection and using qRT-PCR for the detection of DENV genome and serotyping. At 8 to 15 days after the onset of symptoms, the samples are assayed for IgM detection using ELISA. After 15 days, the sera are screened for IgG using ELISA. Dengue infection cannot be excluded in samples that are negative for the NS1 antigen and must be confirmed by IgM/IgG detection. Samples that are negative for dengue will be screened by RT-PCR for chikungunya and Zika virus and other pathogens for differential diagnosis [32]. In Brazil, the recommendation of the Ministry of Health is that all suspected dengue samples must be serotyped. The PCR protocol of Lanciotti et al. [33] is used for confirmation, which is considered the gold-standard protocol for the identification of serotypes by PCR and qPCR techniques.

Several methods based on PCR and qPCR techniques are available in the literature; Table 1 presents the main characteristics of some primers used in these works.

**Table 1:** PCR and qPCR primers

Reference	Sequence	Primer name	Set	Size (bp)	Target
Lanciotti et al. [33] Region (C-prM)	TCAATATGCTGAAACGCGCGAGAAACCG	D1	D1-D2	511	DENV-all
	TTGCACCAACAGTCAATGTCTTCAGGTTC	D2			
	CGTCTCAGTGATCCGGGGG	TS1	D1-TS1	482	DENV-1
	CGCCACAAGGGCCATGAACAG	TS2	D1-TS2	119	DENV-2
	TAACATCATCATGAGACAGAGC	TS3	D1-TS3	290	DENV-3
	CTCTGTTGTCTTAAACAAGAGA	TS4	D1-TS4	392	DENV-4
Chow et al. [34] Region (NS3)	GGRACKTCAGGWTCTCC	upstream	up-down	490	DENV-all
	AARTGIGCYTCRTCCAT	downstream			
Chang et al. [35] Region (NS5)	TTTGAGCATGTCTTCCGTCGTCATCC	Bio-CFD2-4	FUD-CFD2-4 or FUD-CFDJ	838 or 832	DENV-all
	GCATGTCTTCCGTCGTCATCC	Bio-CFDJ9977			
	GATGACACAGCAGGATGGGAC	FUDJ9166			
	GCCTGAACATGCTCTATTGGCT	DEN1-J9243	DEN1-CFD2	761	DENV-1
	TCTTCAAAAGCATTCAGCACCT	DEN2-J9452	DEN2-CFD2	546	DENV-2
	CCCATCCGCTAGAGAAGAAAATTACAC	DEN3-9471	DEN3-CFD2	522	DENV-3
	GGTTTGGCACTTCCCTCCTCTTCTTG	DEN4-9580	DEN4-CFD2	411	DENV-4
Pierre et al. [37] Region (NS5/3'NC)	TGGATGACGACGGAAGACATG	EMF1	EMF1-VD8	600	DENV-all
	GGGTCTCCTCTAACCTCTAG	VD8			
Meiyu et al. [38] Region (NS1)	GACATGGGGTATTGGAT	DJS	DJS-DJA	413	DENV-all
	TCCATCCCATACCTGCA	DJA			
	ATGGATTACCAATATCT	DEN1	DEN1-DJA	262	DENV-1
	GTAAGCTTGAGATGGAC	DEN2	DEN2-DJA	189	DENV-2
	AGCCAAAAGAATGGAAG	DEN3	DEN3-DJA	392	DENV-3
	CTGCATCTGGAAAACTA	DEN4	DEN4-DJA	97	DENV-4
Kuno et al. [39] Region (NS5)	TCAAGGAACTCCACACATGAGATGTACT	FG1-F	FG1-G4	1991	FLAV
	CCAGATGTTCTTWGCCCAYTCTGC	G4-R			
	TACAACATGATGGGAAAGAGAGAGAA	FU1-F	FU1-cFD3	1084	FLAV
	AGCATGTCTTCCGTGGTCATCCA	cFD3-R)			
	GTGTCCCAGCCGGCGGTGTCATCAGC	cFD2 (R)	FU1-cFD2	220	FLAV
Shu et al. [40] Region (C)	CAATATGCTGAAACGCGAGAGAAAC	DN-F	DNF-DNR	170	DENV-all
	CCCCATCTATTCAGAATCCCTGCTC	DN-R			
	CGCTCCATACATCTTGAATGAGC	D1-R	DNF-D1R	190	DENV-1
	AAGACATTGATGGCTTTTGAC	D2-R	DNF-D2R	203	DENV-2
	AAGACGTAAATAGCCCCCGACC	D3-R	DNF-D3R	201	DENV-3
	AGGACTCGCAAAAACGTGATGAATC	D4-R	DNF-D4R	133	DENV-4
Johnson et al. [41] Region (M/E/NS5)	CAAAAGGAAGTCGTGCAATA	DEN-1 F	FAM/BHQ-1	111	DENV1
	CTGAGTGAATTCTCTCTACTGAACC	DEN-1 C			
	CATGTGGTTGGGAGCACGC	DEN-1 probe			
	CAGGTTATGGCACTGTCACGAT	DEN-2 F	HEX/BHQ-1	605	DENV2
	CCATCTGCAGCAACACCATCTC	DEN-2 C			
	CTCTCCGAGAACAGGCCTCGACTTCAA	DEN-2 probe			
	GGACTGGACACACGCACTCA	DEN-3 F	TR/BHQ-2	73	DENV3
	CATGTCTCTACCTTCTCGACTTGTCT	DEN-3 C			
	ACCTGGATGTCGGCTGAAGGAGCTTG	DEN-3 probe			
	TTGTCCTAATGATGCTGGTCG	DEN-4 F	Cy5/BHQ-3	88	DENV4
	TCCACCTGAGACTCCTTCCA	DEN-4 C			
	TTCCTACTCCTACGCATCGCATTCCG	DEN-4 probe			
Ayers et al. [43] Region (NS5)	AATGTACGCTGATGACACAGCTGGCTGGGACAC	FLAVI-1	FLAVI1-FLAVI2	863	FLAV
	TCCAGACCTTCAGCATGTCTTCTGTTGTCATCCA	FLAVI-2			
Chien et al. [44] Region (C-prM)	TCAATATGCTGAAACGCGAGAGAAACCG	mD1	mD1-D2	511	DENV-all
	TTGCACCAACAGTCAATGTCTTCAGGTTC	D2			
	CCCGTAACACTTTGATCGCT	rTS1	mD1-rTS1	208	DENV-1
	CGCCACAAGGGCCATGAACAGTTT	mTS2	mD1-mTS2	119	DENV-2
	TAACATCATCATGAGACAGAGC	TS3	mD1-TS3	288	DENV-3
	TTCTCCCGTTCAGGATGTTC	Rts4	mD1-rTS4	260	DENV-4
Chien et al. [44] Region (NS5)	TACAACATGATGGGAAAGCGAGAGAAAAA	mFU1	mFU1-CFD2	220	FLAV
	GTGTCCCAGCCGGCGGTGTCATCAGC	CFD2			
	TCAGAGACATATCAAAGATTCCAGGGGG	D1P	FAM/BHQ1	220	DENV-1
	AAGAGACGTGAGCAGGAAGGAAGGGGGAGC	D2P	Texas Red/BHQ2	220	DENV-2
	TGAGAGATATTTCCAAGATACCCGGAGGAG	D3P	CY5/BHQ3	220	DENV-3
	TGGAGGAGATAGACAAGAAGGATGGAGACC	D4P	HEX/BHQ1	220	DENV-4
Salles et al. [42] Region (C-prM)	TTTATTTAGAGAGCAGATCTCTG	STD	STD-D2	572	DENV-all
	TTGCACCAACAGTCAATGTCTTCAGGTTC	D2			
	ACGGGTCGACCGTCTTTCAA	STD1	STD1-rTS1	225	DENV-1
	CCCGTAACACTTTGATCGCT	rTS1			
	GCGAAAAACACGCCTTTCAA	STD2	STD2-mTS2	140	DENV-2
	CGCCACAAGGGCCATGAACAGTTT	mTS2			
	ACGGGAAACCGTCTATCAA	STD3	STD3-TS3	302	DENV-3
	TAACATCATCATGAGACAGAGC	TS3			
	GTGGTTAGACCACCTTTCAA	STD4	STD4-rTS4	282	DENV-4
	TTCTCCCGTTCAGGATGTTC	rTS4			

Chow et al. [34] described consensus primers for DENV PCR designed based on motifs conserved within the serine protease and RNA helicase domains encoded by the NS3 genes of dengue and other flaviviruses. However, the authors noted that this method still requires internal primers for the four serotypes of DENV to constitute a nested PCR. This strategy of using consensus primers in PCR depends on DNA sequencing techniques to identify which viral serotype is present in the sample and thus forms a complete molecular diagnostic protocol to characterize the epidemiology of dengue and other flavivirus infections. This protocol is therefore laborious and costly for the identification of DENV serotypes.

Chang et al. [35] presented primers for the nested PCR method for DENV. These primers were designed in the NS5 gene region encoding the viral RNA polymerase because this region is highly conserved in flaviviruses. These authors designed consensus primers and specific internal primers for serotyping, but the amplicons had sizes ranging from 411 to 761 bp, making them inappropriate for qPCR assays [36]. This protocol has a specificity of 89% for DENV serotypes and a sensitivity of 95% for DENV detection. Thus, diagnosis by this protocol still needs improvement.

Pierre et al. [37] proposed a universal single set of consensus primers for flaviviruses. The set was based on conserved elements in the NS5 protein and the 3' untranslated region (3' NC) of flavivirus RNA. Again, this method depends on post-amplification sequencing analysis to characterize the type of flavivirus.

Meiyu et al. [38] described a universal primer set, "DJS (+)/DJA (-)", which was designed for the NS1 gene, which is highly conserved in flaviviruses. Five specific internal primers were also developed based on published NS1 gene sequence data: four of them were for the dengue serotypes (DENV1-4), and one was for Japanese encephalitis virus (JEV). These primers were combined with DJA (-), thus forming five sets of internal primers to produce a hemi-nested assay. The results presented by the authors showed a sensitivity of the PCR assay of 84.6%, necessitating an improvement in the protocol to guarantee greater accuracy in the diagnosis of dengue.

Kuno et al. [39] described a cross-reactive primer set for flavivirus RT-PCR (FU1 and cFD3) with high efficiency to generate 1 kb DNA templates near the 3' terminal end of the flavivirus NS5 gene. The protocol described by the authors aimed to reduce the discrepancy between molecular and serological classifications. The union of these two methods improves discrimination among the members of the genus *Flavivirus* when the RT-PCR amplicon is used for genomic sequencing of the flavivirus.

Shu et al. [40] described a set of group primers and additional serotype-specific primers, which were designed against conserved sequences in the capsid protein (C) gene region and which can be used in RT-PCR and RT-qPCR (SYBR Green) protocols. The results presented by these authors showed a sensitivity of 83% for the SYBR Green-based RT-PCR method, demonstrating that the protocol has a sensitivity below the optimum range (90–110%) determined for the technique and indicating a need for improvement.

Johnson et al. [41] characterized a set of primers in different viral genes, which required three primers to identify each DENV serotype, that targeted the M, E and NS5 protein genes with a TaqMan assay. This method has the advantage of simultaneously identifying all four serotypes, either by using four different probes for each serotype or by using the same probe in separate assays. However, some disadvantages also exist: the cost of the protocol is high, and three primers are required for each viral serotype, two of which are fluorogenic probes for serotyping by RT-qPCR [42].

Ayers et al. [43] demonstrated consensus primers targeting a segment of the NS5 coding region to detect various flaviviruses. This protocol requires the sequencing of amplification products for the confirmation and identification of viral species. Protocol validation was performed, but the authors did not determine the sensitivity of the assay; additional research is required to ensure high sensitivity in further experiments.

Lanciotti et al. [33] described a hemi-nested protocol using primers located in the junction region of the capsid and premembrane (C-prM) genes of DENV; this is the most sensitive method described in the literature [44]. However, the authors reported false-negative PCR results using this protocol due to incompatibility between the dengue viral RNA sequence and the D1, D2 or TS sequences (Table 1). Based on this observation, Chien et al. [44] described modifications of the D1 (mD1) primers and replacement of the primers specific to DENV1, DENV2 and DENV4 by redesigned TS1 (rTS1) and TS4 (rTS4) and modified TS2 (mTS2), leading to primer efficiencies ranging from 72.60 to 89.20% for DENV1, from 70.90 to 96.40% for DENV2, and from 66.90 to 85.80% for DENV4. The TS3 efficiency was similar to that described by Lanciotti et al. [33] for DENV3.

In the Chien et al. [44] study, some modifications were also made to the primers described by Kuno et al. [39]. The consensus primers were redesigned, and internal primers were designed to be used in TaqMan RT-qPCR assays in a nested protocol, taking advantage of the Johnson et al. [41] protocol’s single pair of consensus primers and four oligonucleotides with specific fluorogenic probes to identify each DENV serotype. However, studies have shown that the sensitivity of C-prM is higher (100%) than that of NS5 (91%) [44].

Salles et al. [42] presented modifications to the primers used to detect DENV serotypes, as an update of Chien et al. [44] due to incompatibility between the dengue viral RNA sequence and the protocol. The modified protocol can use less-expensive and more common polymerase enzymes. The hemi-nested protocol was altered by replacing the mD1 primer with STD in the consensus primer region and replacing the mD1 primer with specific internal primers named STD1, STD2, STD3 and STD4 for serotyping. With these modifications, this protocol obtained better yields relative to primer efficiency, from 89.20 to 92.60% (STD1/rTS1) for DENV1, from 96.40 to 101.00% (STD2/mTS2) for DENV2, from 90.10 to 91.10% (STD3/TS3) for DENV3, and from 85.80 to 99.30% (STD4/rTS4) for DENV4.

In addition to the data summarized in Table 1, some commercial PCR kits exist, such as the CDC’s DENV1-4 RT-PCR assay kit and those from Geno-Sen, LifeRiver, RealStar and Simplexa. However, these kits are expensive and do not provide 100% sensitivity, making it difficult to use them in the Brazilian Unified Health System [41, 45].

## Prevention and control of dengue in Brazil

The French pharmaceutical company Sanofi-Aventis, the world’s leading vaccine producer, has launched a commercial vaccine for the prevention of dengue fever called Dengvaxia. This pentavalent chimeric vaccine is derived from all four serotypes of DENV and YFV [46]. This vaccine has been licensed in several American countries: Mexico, Brazil, El Salvador, Costa Rica, Paraguay, Guatemala and Peru [47]. ANVISA (the Brazilian National Health Surveillance Agency) licensed Dengvaxia for Brazil in 2015 [48]. This vaccine presents some serious problems that call into question its full effectiveness [47]. In addition, this vaccine has a high cost, and further studies are needed to prove its efficacy [47]. In November 2017, ANVISA released a note recommending the non-use of the Dengvaxia vaccine in Brazil [49]. This study was based on the possibility that the Dengvaxia vaccine might increase the risk of severe disease in people who had never been exposed to DENV before. Since then, this vaccine has not been considered as a potential prevention tool. The Philippine Health Ministry halted Dengvaxia immunizations due to 14 deaths of children that were associated with vaccination [50]. In Brazil, the government decided to use Dengvaxia to vaccinate only individuals who are already seropositive [49].

In Brazil, all three government spheres (federal, state and municipality) share responsibility for dengue control. The federal level provides guidelines for vector control, allocates resources to the states and purchases insecticides and equipment, such as vehicles mounted with an ultra-low-volume sprayer to support chemical control. The states assist and supervise municipalities, acquire consumables and small equipment, such as nylon nets and lids for water tanks or mosquito traps, and gather information about the municipalities to notify the Health Ministry. The municipality is responsible for operations such as management of vector control professionals and actions, following central-level recommendations. In practice, this shared responsibility can reduce the efficiency of vector control; for example, decision-making processes can be bureaucratic and time-consuming [51].

The prospects of controlling dengue disease are not promising. The number of dengue cases increases in proportion with factors such as deforestation, poor sanitation and climate change [51]. Three out of four Brazilian municipalities are heavily infested with the mosquito *Ae. aegypti*. Reducing the density of *Ae. aegypti*, the main link in the transmission chain, remains a challenge. Under the coordination of the National Program for Dengue Control (PNCD) of the Ministry of Health, an International Meeting for Implementation of New Alternatives for *Ae. aegypti* Control was held in Brazil in February 2016 [52, 53]. Some vector control initiatives have been conducted. These efforts include re-emphasizing insecticide use, but most mosquito populations are resistant [52, 53]. Other initiatives, such as sterilizing mosquitoes by genetic modification or irradiation or even infecting mosquitoes with *Wolbachia*, have so far shown no success in vector control [52, 53]. Another form of prevention consists of an ongoing battle against water accumulation, which is conducive to the reproduction of the mosquitoes that transmit the disease. For this reason, periodic epidemiological surveillance and population awareness are necessary to control the reproduction of the mosquito vector by promoting actions such as the removal of water that accumulates in breeding sites [54]. This initiative can be efficient in the home, but much water accumulates in peridomicile environments in Brazil due to the precarious system of water supply, sewage and solid waste collection among others. These conditions favor the proliferation of the *Ae. aegypti* mosquito in cities, making it difficult or almost impossible to combat the vector [55].

Even with the government investing more than half a billion dollars each year in mosquito control, there has been no reduction in vector density that could limit or reduce the spread of dengue in a sustained way [56].

Without vaccines, effective drugs, or sensitive diagnostic tests, the only available response to reduce disease severity and case fatality is clinical management through enhanced care supported by accessible, sensitive and specific useful diagnostic tests. These tools will help identify warning signs for severe disease and evidence-based criteria for the standardization of treatment procedures [57]. During epidemics, Brazil’s public health initiatives are aimed at increasing awareness of the signs and symptoms of the disease to facilitate the earlier arrival of health services to allow the early diagnosis and treatment of severe forms of dengue.

## Conclusions

Without adequate prevention and treatment, the number of reported cases of dengue has increased in recent years in Brazil and the Americas. Considering these facts, the population is dependent on methods of prevention and the treatment of symptoms. For this reason, the Secretariat of Health Surveillance (SHS) must collect information from the data provided by sentinel laboratories, even if not all cases are confirmed due to diagnostic inefficiency and some must be discarded. Studies are necessary to increase the performance of the available diagnostic methods to guarantee accurate notifications by state health departments and municipalities. Finally, it is important to continue to support the research for more effective diagnosis and treatment of dengue and also for new methodologies of vector control and disease prevention.

## Additional file

Additional file 1: **Table S1.** Cases of dengue. Brazil, major regions and federated units, 1990 to 2017. (DOCX 20 kb)

## Abbreviations

ANVISA: National Health Surveillance Agency
C: Capsid protein
CFR: Case fatality rate
CHIKV: Chikungunya virus
DENV: Dengue virus
DENV1: Dengue virus serotype 1
DENV2: Dengue virus serotype 2
DENV3: Dengue virus serotype 3
DENV4: Dengue virus serotype 4
DF: Dengue fever
DHF: Dengue hemorrhagic fever
DSS: Dengue shock syndrome
E: Envelope proteins
ECI: Evandro Chagas Institute
EW: Epidemiological week
FU: Federative Units
IgG: Immunoglobulin G
IgM: Immunoglobulin M
JEV: Japanese encephalitis virus
LACENS: Brazilian State Central Reference Laboratories
M: Membrane protein
NS1, NS2A, NS2B, NS3, NS4A, NS2K, NS4B and NS5: Non-structural proteins
PAHO: Pan-American Health Organization
PCR: Polymerase chain reaction
PNCD: National Program For Dengue Control
qPCR: quantitative PCR
RT-PCR: Reverse transcription PCR
RT-qPCR: RT-quantitative PCR
SHS: Secretariat of Health Surveillance
SLEV: Saint Louis encephalitis virus
TBEV: Tick-borne encephalitis virus
WHO: World Health Organization
WNV: West Nile virus
YFV: Yellow fever virus
ZIKV: Zika virus
