# Supplementary material for: History, epidemiology and diagnostics of dengue in the American and Brazilian contexts: a review
Source: Parasit Vectors. 2018 Apr 24;11:264. doi: 10.1186/s13071-018-2830-8 (PMC5937836; doi:10.1186/s13071-018-2830-8)
Supplement: Supplementary file 1 — Table S1. Cases of dengue. Brazil, major regions and federated units, 1990 to 2017. (DOCX 20 kb) [file 13071_2018_2830_MOESM1_ESM.docx]

| **Federative Units per Region** | **2011** | | **2012** | | **2013** | | **2014** | | **2015** | | **2016** | | **2017** | |
| --- | --- | --- | --- | --- | --- | --- | --- | --- | --- | --- | --- | --- | --- | --- |
|  | **Number cases** | **Incidence (/100,000 hab.)** | **Number cases** | **Incidence (/100,000 hab.)** | **Number cases** | **Incidence (/100,000 hab.)** | **Number cases** | **Incidence (/100,000 hab.)** | **Number cases** | **Incidence (/100,000 hab.)** | **Number cases** | **Incidence (/100,000 hab.)** | **Number cases** | **Incidence (/100,000 hab.)** |
| **Region North** | 119,398 | 612,4 | 42,158 | 190.0 | 49,574 | 287.0 | 48,376 | 278.2 | 31,411 | 197.6 | 39,011 | 212.9 | 22,660 | 128.0 |
| Rondônia | 3,219 | 154.1 | 3,292 | 80.4 | 8,732 | 499.4 | 1,985 | 112.5 | 2,211 | 125.5 | 7,769 | 432.3 | 2,460 | 137.6 |
| Acre | 18,865 | 2,376.2 | 2,393 | 279.6 | 2,568 | 325.0 | 28,269 | 3,547.4 | 5,317 | 736.5 | 2,335 | 264.2 | 2,124 | 260.1 |
| Amazonas | 61,986 | 1,612.4 | 5,167 | 86.4 | 17,832 | 460.3 | 6,661 | 171.4 | 3,792 | 106.6 | 8,125 | 186.7 | 3,984 | 99.6 |
| Roraima | 1,451 | 127.9 | 1,874 | 182.5 | 945 | 190.2 | 1,123 | 225.0 | 1,097 | 219.1 | 246 | 40.6 | 316 | 61.5 |
| Pará | 19240 | 181.1 | 16,246 | 148.0 | 9,166 | 113.1 | 4,496 | 55.4 | 7,803 | 108.7 | 10,852 | 129.0 | 7,813 | 94.4 |
| Amapá | 2,803 | 323.5 | 1,569 | 29.1 | 1,708 | 227.5 | 2,190 | 290.6 | 3,297 | 421.8 | 1,798 | 228.9 | 886 | 113.3 |
| Tocantins | 11,834 | 337.1 | 11,617 | 837.7 | 8,596 | 574.3 | 3,652 | 241.0 | 7,894 | 594.4 | 7,886 | 500.0 | 5,077 | 331.2 |
| **Region Northeast** | 195,385 | 217.1 | 222,913 | 195.3 | 152,357 | 271.2 | 89,935 | 159.4 | 327,212 | 554.4 | 324,815 | 556.8 | 86,386 | 151.8 |
| Maranhão | 11,777 | 88.8 | 5,325 | 45.3 | 3,588 | 52.4 | 2,652 | 38.3 | 7,943 | 109.5 | 24,167 | 339.8 | 7,049 | 101.4 |
| Piauí | 10,060 | 175.1 | 12,255 | 156.1 | 4,987 | 156.1 | 7,657 | 239.5 | 7,646 | 238.5 | 5,242 | 161.2 | 5,184 | 161.4 |
| Ceará | 63,206 | 539.3 | 54,831 | 203.6 | 30,219 | 341.7 | 22,756 | 256.3 | 63,116 | 718.3 | 50,897 | 550.5 | 40,604 | 453.0 |
| Rio Grande do Norte | 23,171 | 420.1 | 28,778 | 324.7 | 18,905 | 554.6 | 11,498 | 335.5 | 22,700 | 660.2 | 57,508 | 1,629.4 | 7,311 | 210.4 |
| Paraíba | 12,602 | 214.8 | 8,771 | 67.1 | 13,466 | 341.4 | 5,625 | 142.3 | 23,426 | 552.0 | 35,688 | 882.3 | 3,837 | 95.9 |
| Pernambuco | 22,101 | 102.5 | 31,799 | 311.4 | 7,985 | 86.1 | 10,488 | 112.1 | 110,899 | 1,107.2 | 65,152 | 632.6 | 9,043 | 96.1 |
| Alagoas | 8,909 | 141.3 | 28,123 | 207.2 | 11,296 | 340.1 | 13,186 | 396.1 | 27,130 | 718.7 | 17,301 | 535.4 | 2,930 | 87.2 |
| Sergipe | 3,927 | 54.6 | 4,558 | 184.4 | 801 | 36.1 | 2,246 | 100.6 | 9,141 | 381.2 | 3,509 | 148.7 | 609 | 26.9 |
| Bahia | 39,612 | 160.0 | 48,473 | 200.9 | 61,110 | 404.0 | 13,827 | 91.3 | 55,211 | 354.0 | 65,351 | 432.5 | 9,819 | 64.3 |
| **Region Southeast** | 361,350 | 291.1 | 251,738 | 148.6 | 918,226 | 1,078.8 | 311,639 | 365.1 | 1,047,279 | 1,205.7 | 858,273 | 981.5 | 59,601 | 69.0 |
| Minas Gerais | 40,343 | 131.9 | 29,456 | 71.5 | 416,252 | 2,007.6 | 58,177 | 279.9 | 192,779 | 913.4 | 528,441 | 2,489.6 | 28,779 | 137.1 |
| Espiríto Santo | 40,336 | 630.9 | 11,961 | 158.2 | 67,995 | 1,750.2 | 18,879 | 484.4 | 35,441 | 893.1 | 41,736 | 1,044.1 | 7,019 | 176.6 |
| Rio de Janeiro | 165,787 | 665.7 | 181,169 | 501.3 | 213,058 | 1,294.3 | 7,717 | 46.6 | 73,437 | 417.1 | 85,200 | 511.5 | 10,592 | 63.7 |
| São Paulo | 114,884 | 192.6 | 29,152 | 47.7 | 220,921 | 501.7 | 226,866 | 513.7 | 745,622 | 1,665.7 | 202,896 | 443.0 | 13,211 | 29.5 |
| **Region South** | 35,978 | 108.3 | 4,772 | 12.1 | 66,903 | 230.6 | 22,988 | 79.1 | 51,681 | 193.6 | 72,650 | 238.1 | 4,678 | 15.9 |
| Paraná | 35,438 | 280.1 | 4,508 | 29.5 | 66,100 | 596.5 | 22,701 | 204.5 | 45,542 | 448.7 | 64,305 | 550.6 | 4,195 | 37.3 |
| Santa Catarina | 177 | 1.6 | 94 | 1.3 | 358 | 5.3 | 134 | 2.0 | 4,441 | 69.4 | 5,150 | 72.9 | 256 | 3.7 |
| Rio Grande do Sul | 363 | 2.8 | 170 | 1.3 | 445 | 4.0 | 153 | 1.4 | 1,698 | 16.0 | 3,195 | 27.8 | 227 | 2.0 |
| **Region Midwest** | 51,942 | 226.1 | 68,010 | 209.7 | 265,456 | 1,744.2 | 116,169 | 760.1 | 231,105 | 1,451.9 | 205,786 | 1,349.5 | 78,729 | 502.7 |
| Mato Grosso do Sul | 8,510 | 245.8 | 9,202 | 187.0 | 78,958 | 3,014.1 | 3,423 | 128.4 | 32,241 | 1,068.4 | 44,814 | 1,689.1 | 2,112 | 78.7 |
| Mato Grosso | 6,146 | 116.6 | 32,856 | 454.7 | 35,190 | 1,091.4 | 7,160 | 221.1 | 21,583 | 627.2 | 19,940 | 603.3 | 8,977 | 271.6 |
| Goiás | 33,960 | 338.2 | 24,517 | 170.4 | 139,357 | 2,136.3 | 93,929 | 1,434.1 | 167,427 | 2,500.6 | 123,195 | 1,918.0 | 63,430 | 947.3 |
| Distrito Federal | 3,325 | 74.8 | 1,435 | 33,7 | 11,951 | 419.0 | 11,657 | 408.3 | 9,854 | 337.9 | 17,837 | 593.4 | 4,210 | 141.4 |
| **Brazil** | 764,032 | 266,2 | 589,591 | 149,9 | 1,452,489 | 716.2 | 589,107 | 289.4 | 1,688,688 | 813.1 | 1,500,535 | 719.9 | 252,054 | 122.3 |

**Additional file 1: Table S1**. Cases of dengue. Brazil, major regions and federated units, 1990 to 2017.

Source: SES / SINAN (SINAN: from 1999), From the Sinan data, all the cases were tabulated, except those discarded.

* Updated 2/8/2018. Data subject to change.
